# Anxiety, Depression, and Fear of COVID-19 and Its Association With Psychosocial Factors Among the Employees of a Malaysian Public University During Movement Control Order

**DOI:** 10.7759/cureus.73676

**Published:** 2024-11-14

**Authors:** Asma Assaedah Mahmud, Fatimah Zahra Mohamad Rom, Rosnadia Suain Bon, Maizatullifah Miskan, Mainul Haque

**Affiliations:** 1 Psychiatry, Faculty of Medicine and Defence Health, National Defence University of Malaysia, Kuala Lumpur, MYS; 2 Medicine, Faculty of Medicine and Defence Health, National Defence University of Malaysia, Kuala Lumpur, MYS; 3 Primary Care Medicine, Faculty of Medicine and Health Defence, National Defence University of Malaysia, Kuala Lumpur, MYS; 4 Pharmacology and Therapeutics, Faculty of Medicine and Defence Health, National Defence University of Malaysia, Kuala Lumpur, MYS

**Keywords:** defence university, emergence of coronavirus, employees, gad- 7, mental health, phq-9, psychology, social factors, universities, young and elderly people

## Abstract

Background: This cross-sectional study among the employees of the National Defense University of Malaysia (NDUM) assessed the levels of depression, anxiety, and fear related to COVID-19 and its association with psychosocial factors. It was conducted during the strict lockdown period imposed by the Malaysian government due to the COVID-19 pandemic.

Methods: A stratified sampling method was used, and 277 employees were randomly selected to participate in the study. Generalized anxiety disorder (GAD-7) was used to measure anxiety symptoms. Patient health questionnaire (PHQ-9) was utilized to assess depression. The fear of COVID-19 (FOC) was calculated using the Fear of COVID-19 Scale (FCV), while the psychosocial factors affecting psychological impact were measured using a self-generated questionnaire.

Results: The study obtained a response rate of 46.2% (n=128). Most respondents were female, married, permanent employees, and from the non-academic group. Depressive and anxiety symptoms were reported by 47% (n=55) and 32.5% (n=38) of the respondents, respectively. Statistically, a higher level of fear related to COVID-19 was found among non-academic employees (p=0.015) and those with permanent employment status (p=0.030). Anxiety was significantly correlated with depression (r=0.70, p≤0.001), while no correlations were found between these factors and fear related to COVID-19. Taking over school lessons was the most troubling factor that affected the respondents' level of distress, followed by working from home and worrying about their family member's health.

Conclusions: This study identified significant psychological effects of the pandemic on university employees, with anxiety and depression being notably correlated. While the generalizability of the findings is limited due to a low response rate, several key psychosocial distress factors were identified. These findings emphasize the necessity of addressing psychosocial factors to mitigate the mental health impact of pandemics. Further research with a higher response rate is required to confirm these findings and to design targeted interventions to support affected employees.

## Introduction

The emergence of COVID-19 has substantially affected global mental health. The pandemic has been associated with heightened levels of stress, anxiety, depression, and other mental health issues, driven by factors such as social isolation, economic uncertainty, health-related fears, and disruptions to daily routines. The turbulence caused by the virus can be felt at individual, collective, and organizational levels. Most studies found between 23-40% of the general population suffered from moderate to severe psychological distress such as depression, anxiety, stress, or post-traumatic disorder symptoms during the earlier stage of the pandemic [1-4]. The highest level of psychological distress was seen among young and older adults, women, those from socially or economically disadvantaged backgrounds, and those who lost their jobs during the lockdown [5-7].

The psychological distress among the general population may be attributed to various factors, such as individual's perception regarding safety and health during a pandemic, threat and risk of contagion, excessive information versus the unknown, living in quarantine and confinement, limited social support system, and financial loss [8]. Meanwhile, several studies among non-healthcare workers, including those in higher learning institutions, found a significant level of anxiety and depressive symptoms attributed to numerous reasons, for instance, job insecurities, working in isolation, abrupt changes in the working environment, and also difficulties related to distance education [9-12]. Another study at Michigan State University found that 27.6% of university staff reported having various forms of psychological distress during the different phases of the lockdown [13].

Before the pandemic, the mental health landscape among educators in Malaysian higher learning institutions showed alarming trends, especially regarding perceived symptoms of depression, anxiety, and stress. A local study involving staff at a Malaysian public university found that the prevalence of perceived symptoms among respondents was 28.7% for depression, 50.1% for anxiety, and 14.8% for stress [14]. A systematic review further revealed that the prevalence of stress was reported solely among educators at higher learning institutions, including both private colleges and public universities, with rates varying from 5.5% to 25.9%. Additionally, 5.5% of academicians in private universities reported experiencing burnout [15].

During the pandemic, limited data was reported on the psychological impact of COVID-19 among the Malaysian population, particularly during the strict lockdown measures known as the Movement Control Order (MCO) [16]. A general population study conducted in Borneo, Malaysia, found that higher levels of fear of COVID-19 (FOC), as well as depression, anxiety, and stress, were reported among female respondents and those under 25 years old [17]. Another study in Malaysia concluded that individuals financially affected by the pandemic, those who consumed alcohol, and those with high levels of FOC were associated with more considerable psychological distress [18]. Most local studies focused on the psychological impact on university staff and students, while the potential psychosocial stressors have not been fully explored [19-22]. Several issues remain poorly understood, particularly regarding the role of social background, employment status, and psychosocial stressors in individuals' psychological responses to the pandemic.

Objectives of the study

Therefore, this study aimed to examine the level of anxiety, depression, and FOC among National Defense University of Malaysia (NDUM) employees, to assess the correlation between anxiety, depression, and fear related to COVID-19, and to explore anxiety, depression, and fear and its association with psychosocial factors during the pandemic.

## Materials and methods

**Figure 1:**
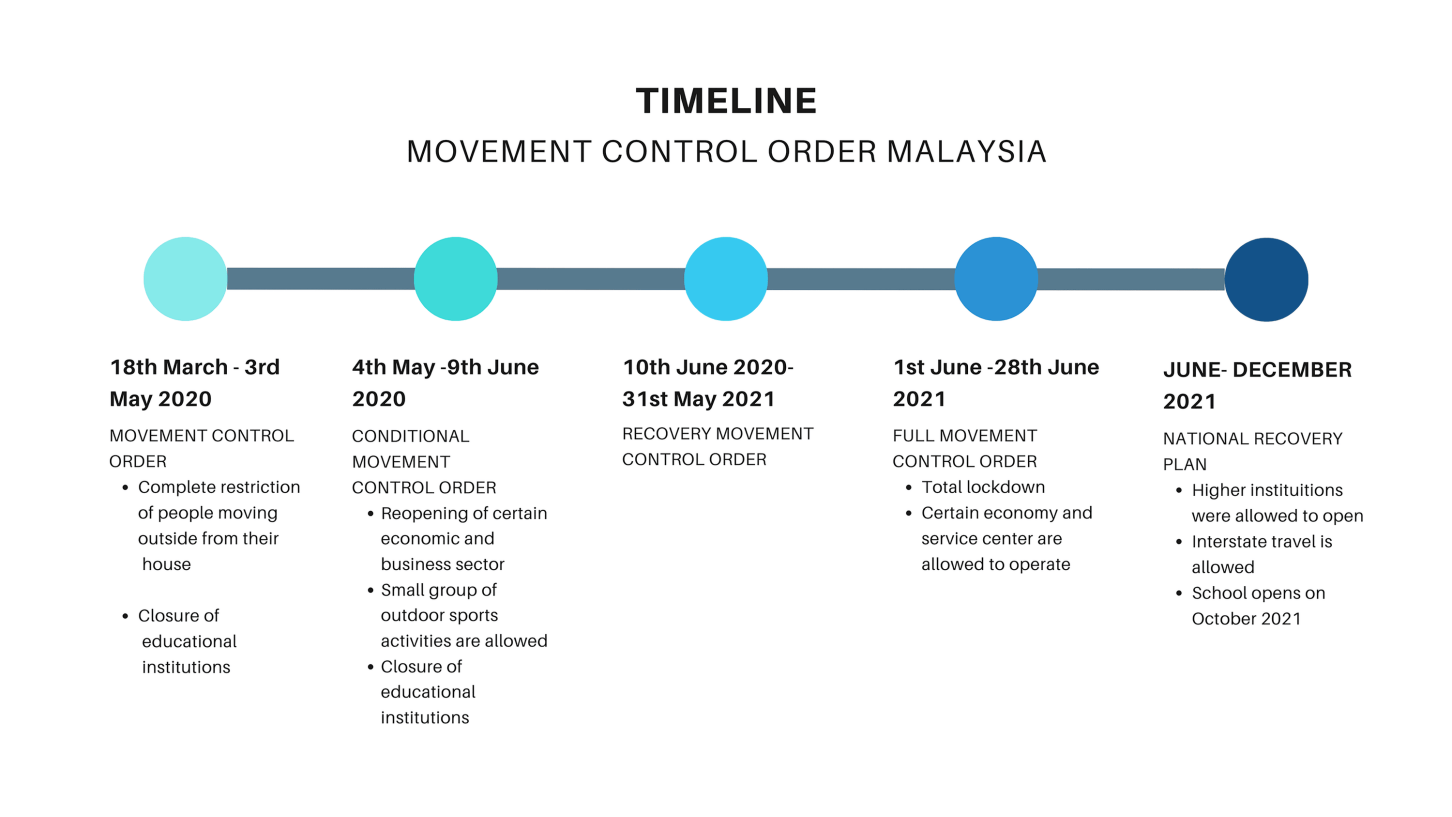
MCO Notes: This figure has been drawn utilizing the Canva premium version (https://www.canva.com/) [25]. Image Credit: Fatimah Zahra Mohamad Rom. MCO: Movement Control Order

This study was a cross-sectional online survey conducted at the NDUM. Data collection occurred from July to September 2021 during the National Recovery Plan by the Malaysian government mandated by the MCO [23]. During this phase, the social and educational sectors remained closed (Figure 1) [24].

The study population was drawn from the NDUM employee cohort, consisting of 983 employees, which includes 382 academicians and 601 non-academic staff, reflecting a ratio of 1 academician to 1.57 non-academics. The inclusion criteria were NDUM employees who could read and write in English or Malay. The sample size for a known population was calculated using the online sample size calculator [26]. Based on a 95% confidence interval and a 5% margin of error, a minimum sample size of 277 was required for adequate statistical power.

**Figure 2:**
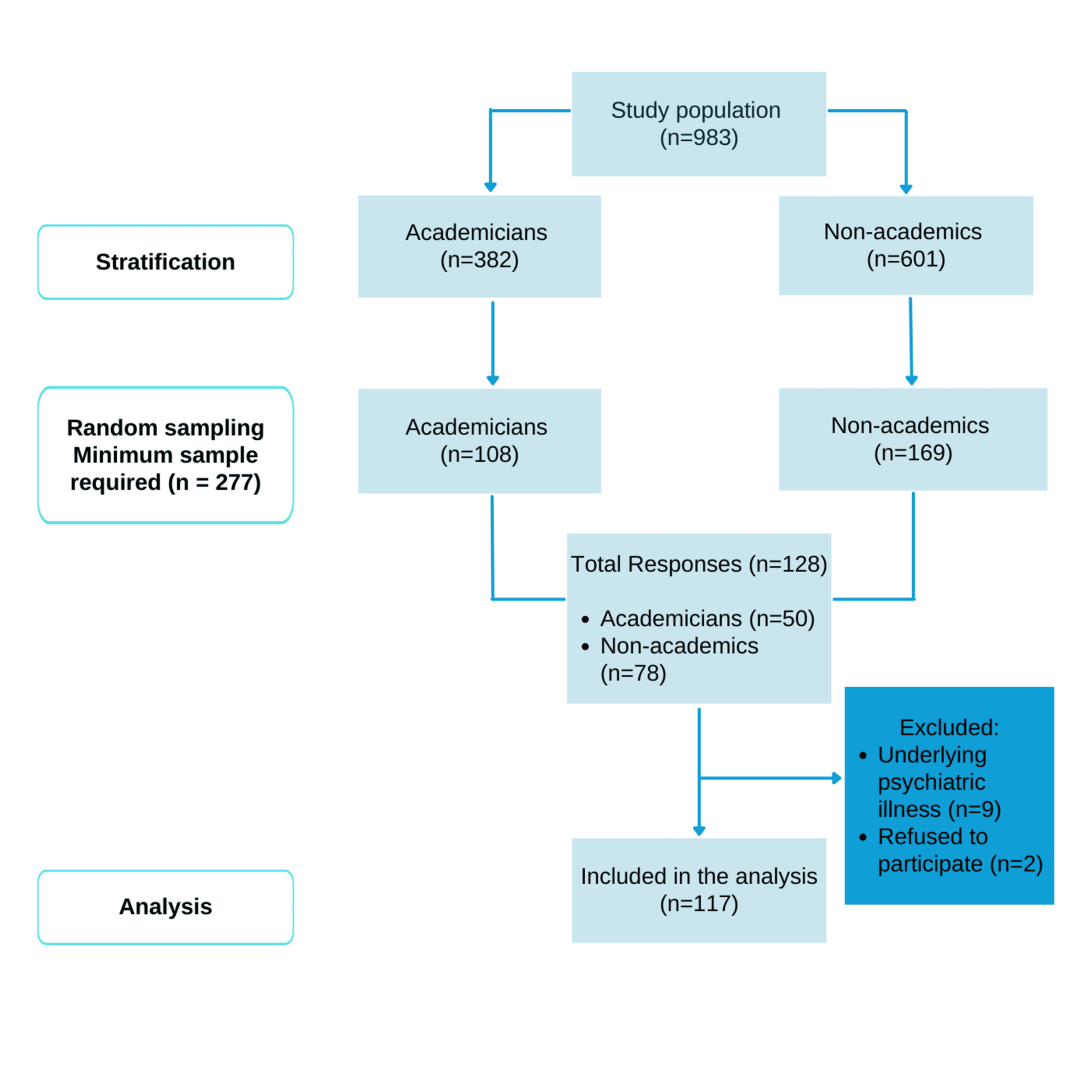
Methods of this study Notes: This figure has been drawn utilizing the Canva premium version (https://www.canva.com/) [25] Image Credit: Fatimah Zahra Mohamad Rom.

A stratified sampling method used employment categories (academicians and non-academics) to ensure equal representation. Randomization lists for both groups were generated using an online random sequence generator [27]. Next, participants were randomly selected based on a ratio of 1 academician to 1.57 non-academicians, designed to reflect the population structure. All selected participants were invited to access the electronic survey via Google Forms sent to their work emails, with follow-up reminders issued to enhance participation rates. Participants who had underlying psychiatric illnesses were excluded during the analysis process. The flow chart of this study is illustrated in Figure 2. 

Dependent variables

Depression

A validated English and Malay version of the Patient Health Questionnaire-9 (PHQ-9) was utilized to evaluate depressive symptoms over the past two weeks (Appendix 1) [28,29]. This self-rated questionnaire effectively assesses a range of depressive symptoms, including diminished interest in activities, persistent feelings of sadness or hopelessness, sleep disturbances, fatigue, changes in appetite, feelings of worthlessness, difficulty concentrating, psychomotor agitation or retardation, and thoughts of self-harm. The PHQ-9 demonstrates strong reliability, with internal consistency coefficients (Cronbach's alpha) typically ranging from 0.86 to 0.89 across diverse populations. Each of the nine items is rated on a 0-3 scale (0 = not at all, 1 = several days, 2 = more than half of the days, 3 = nearly every day), yielding a total score that ranges from 0 to 27. The scoring thresholds indicate severity levels: 0-4 for minimal or no depression, 5-9 for mild depression, 10-14 for moderate depression, 15-19 for moderately severe depression, and 20-27 for severe depression. A cut-off point of 10 follows the initial validation study from Kroenke et al., which had a sensitivity of 88% and a specificity of 88% for detecting major depressive disorders [29].

Anxiety

Spitzer et al. designed a generalized anxiety disorder questionnaire (GAD-7), a self-rated tool to assess anxiety symptoms in the last two weeks (Appendix 2) [30]. The Malay version of the GAD-7 was already validated and was used in this study [31]. It has demonstrated excellent reliability, with Cronbach's alpha coefficient ranging from 0.89 to 0.94, indicating high internal consistency [29]. The GAD-7 comprises seven items that respondents rate on a scale from 0 to 3 (0 = not at all, 1 = several days, 2 = more than half the days, 3 = nearly every day). The total score can range from 0 to 21, with established thresholds for anxiety severity: 0-4 indicates minimal anxiety, 5-9 reflects mild anxiety, 10-14 suggests moderate anxiety, and 15-21 signifies severe anxiety. The items assess a range of anxiety-related symptoms, including feelings of nervousness, difficulty in controlling worry, excessive worry about various issues, trouble relaxing, restlessness, irritability, and a persistent sense of impending doom. This scoring system not only facilitates the classification of anxiety severity but also provides essential insight into the individual's mental health status, enabling more targeted interventions.

Fear of COVID-19

Fear related to COVID-19 was measured using the seven-item Fear of COVID-19 Scale (FCV-19S), a validated self-rated tool designed to assess the emotional response and fear associated with the pandemic [32]. Each item is scored on a five-point Likert scale, ranging from 1 (Strongly Disagree) to 5 (Strongly Agree), with higher scores indicating greater fear. The total score ranges from 7 to 35, and following the cut-off point established by Rahman et al., scores between 7-21 indicate low levels of fear, while 22-35 reflect high levels of fear [31]. The FCV-19S has demonstrated good reliability, with reported Cronbach's alpha coefficients ranging from 0.82 to 0.90, indicating strong internal consistency across studies [33]. The scale covers a range of fear-related responses, including worry, physical symptoms (such as increased heart rate), and emotional distress. Items address concerns like becoming anxious about COVID-19, feeling uncomfortable thinking about the pandemic, fearing loss of life due to the virus, and experiencing physiological reactions like sweating or sleep disturbances when thinking about COVID-19.

Independent variables

Demographic Characteristics

The demographics included are gender, age, marital status, years working in NDUM, employment status, and the available options for employees to work from the office or remotely. Additionally, various types of remote work arrangements were documented in the study.

Psychosocial Factors

Based on literature reviews, the researchers found several psychosocial factors that were considered to be significant in influencing the employees' psychological well-being, which are: worries about their health (F1), concerns about their family's health (F2), financial worries (F3), difficulties to adapt to new norms (F4), uncertainties of the future (F5), increase conflict with people close to them (F6), being in quarantine (F7), working from home (F8) and taking over school lessons (F9) [9-11, 32-36]. The responses were recorded based on yes, no, or not applicable for each factor. Before the questionnaire was distributed, a pilot study was performed to validate the face of the questionnaire. Feedback was obtained concerning the understanding of the questionnaire, and appropriate changes were made based on the responses.

Data analysis

Data analysis was performed using IBM Statistical Package for Social Sciences (SPSS) Statistics version 26 (New York, USA). Categorical data are reported as numbers and percentages. Quantitative data with normal distribution were expressed as (mean ± standard deviation), and those that did not show normal distribution were expressed as median minimum and maximum values. After conducting a Kolmogrov-Smirnov test, the result indicated that the data were not normally distributed. Consequently, a non-parametric test was used to measure the outcome. Mann Whitney U test and Kruskal Wallis test were applied to compare depression, anxiety, and fear related to COVID-19 with the demographic data. The correlation between depression, anxiety, and FOC was analyzed using the Spearman correlation test. A p-value of less than 0.05 indicates significant findings.

Ethical approval

This study obtained ethical approval from the Institutional Review Board, Universiti Pertahanan Nasional Malaysia (National Defence University of Malaysia), with the number series SF0108-UPNM/2021/SF/SKK/2, dated July 2021. The participants were duly informed about the study aims and procedures and signed an online informed consent form. We employed data encryption and limited access to their personal information to protect participants' anonymity and confidentiality.

## Results

Among the 277 participants invited to the study, 128 completed the survey, resulting in a response rate of 46.2%. The response rates were similar across the two groups: 44.4% (n = 50) of academicians and 46% (n = 78) of non-academic staff responded to the invitation. Ultimately, only 117 responses were eligible for inclusion in the final data analysis. Nine participants were excluded due to underlying psychiatric conditions, and two declined to provide consent.

Table 1 represents the demographic characteristics of the study participants. Most were female, married, permanent employees, and from the non-academic group. During the study period when the strict MCO was mandated, 50 (42.7%) participants were allowed to work six to seven days per week at home.

**Table 1:** Demographic characteristics of the study participants WFH: Work from home; MCO: Movement Control Order

Variables	Descriptions	N = 117 (%)
Mean age (with SD)	-	39.62 years (7.19)
Gender	Male	44 (37.6)
	Female	73 (62.4)
Marital status	Single	18 (15.4)
	Married	99 (84.6)
Years of working in NDUM (Mean)	-	10.89 years
Employment status	Contract	11 (9.4)
	Permanent	106 (90.6)
Employment category	Academic	45 (38.5)
	Non-academic	72 (61.5)
WFH option during MCO	No (Working full time in office)	8 (6.8)
	Yes (1-3 days/week)	26 (22.2)
	Yes (4-5 days/week)	33 (28.2)
	Yes (6-7 days/week)	50 (42.7)

**Figure 3:**
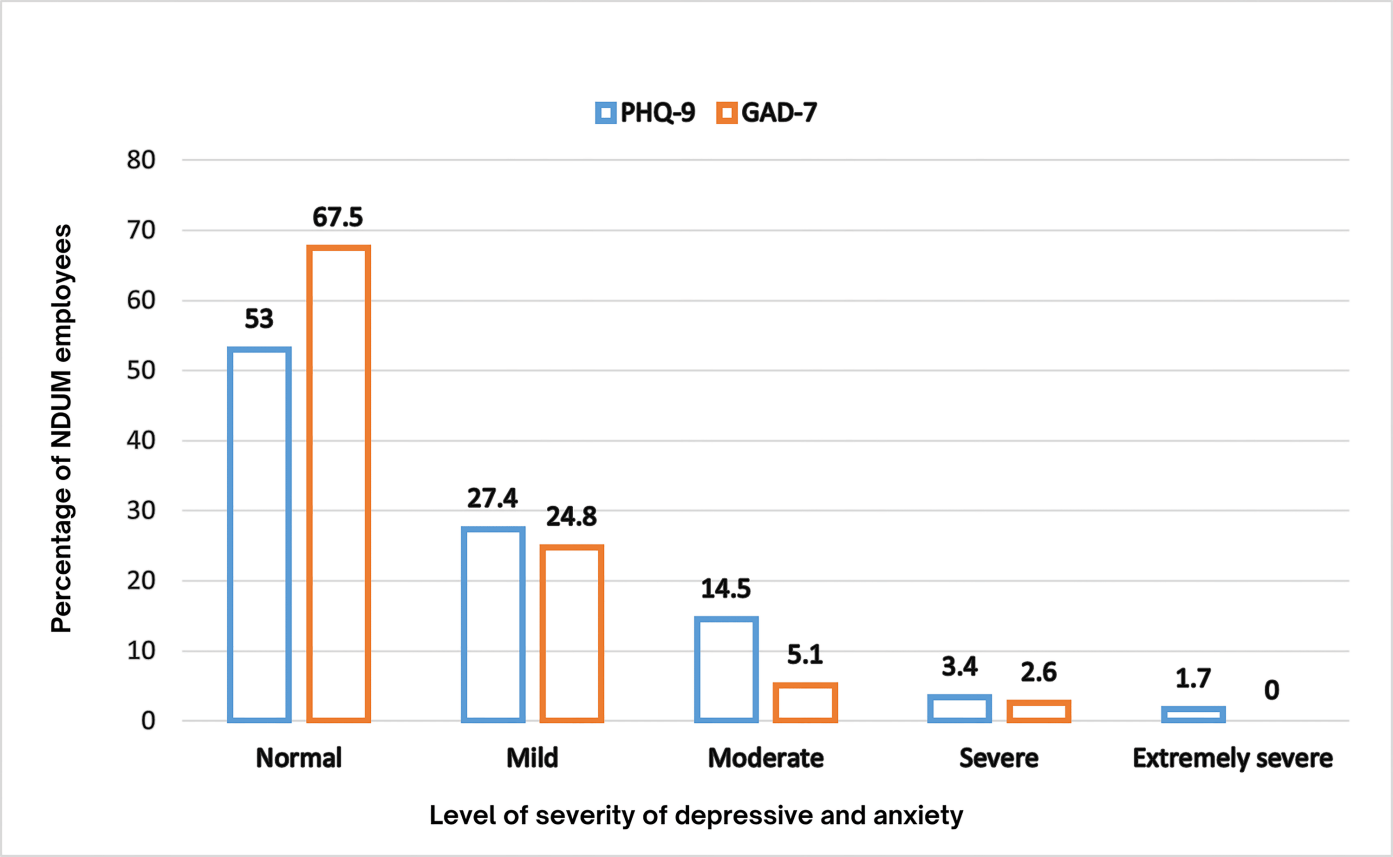
Percentage of depression and anxiety symptoms among NUDM employees by the level of severity This figure has been drawn utilizing the Canva premium version (https://www.canva.com/) [25] Image Credit: Fatimah Zahra Mohamad Rom. NUDM: National Defence University of Malaysia; PHQ-9: Patient Health Questionnaire-9; GAD-7: General Anxiety Disorder-7

Figure 3 shows the percentage of NDUM employees according to the level of severity of the depressive and anxiety symptoms. Depressive symptoms were reported by 47% of participants (n = 55), while 32.5% (n = 38) experienced anxiety symptoms. Furthermore, 37.6% of participants (n = 44) reported experiencing heightened fear associated with COVID-19. Using the Spearman rank correlation test, a positive correlation was found between anxiety and depression (r = 0.70, p≤0.001). In contrast, no correlation was found between anxiety and fear related to COVID-19 (r = 0.136, p = 0.145) and depression with FOC (r = - 0.039, p = 0.679). 

Table 2 shows the analysis of demographic characteristics and anxiety, depression, and fear related to COVID-19 using the Mann-Whitney and Kruskal-Wallis tests. Statistically, higher levels of fear associated with COVID-19 were found among non-academic employees (p = 0.015) and those with permanent employment status (p = 0.030).

**Table 2:** Comparison of median scores for depression, anxiety, and FOC across sociodemographic variables ^a^ Mann-Whitney test; ^b^ Kruskal-Wallis test; * p<0.05 statistically significant PHQ: Patient health questionnaire; GAD: General anxiety disorder; FOC: Fear of COVID-19; WFH: Work from home; MCO: Movement Control Order

Variables	Categories	Number (N)	Median PHQ score (Min-Max)	p-value	Median GAD score (Min-Max)	p-value	Median FOC score (Min-Max)	p-value
Gender^a^	Male	44	3 (0-20)	0.273	4 (0-23)	0.832	20 (7-34)	0.311
	Female	73	4 (0-19)		3 (0-18)		21 (7-32)	
Age^a^	21-40 years	75	4 (0-23)	0.264	3 (0-20)	0.196	20 (7-32)	0.722
	41-60 years	42	3.5 (0-11)		2 (0-13)		21 (9-34)	
Marital status^a^	Single	18	4 (0-23)	0.077	3 (0-20)	0.416	21 (7-34)	0.386
	Married	99	5.5 (0-19)		3 (0-18)		20 (7-29)	
Employment category^a^	Academic	45	5 (0-14)	0.806	3 (0-9)	0.535	19 (7-29)	0.015*
	Non-academic	72	4 (0-23)		2.5 (0-20)		21 (7-34)	
Employment status^a^	Contract	11	12 (0-19)	0.078	4 (0-18)	0.544	16 (7-24)	0.030*
	Permanent	106	4 (0-23)		3 (0-20)		21 (7-34)	
Years of working^a^	1-10 years	41	4 (0-19)	0.713	3 (0-18)	0.531	20 (7-29)	0.414
	11-20 years	76	4 (0-23)		3 (0-20)		21 (7-34)	
WFH option during MCO^b^	No	8	4 (0-19)	0.644	5.5 (0-16)	0.572	19.5 (9-34)	0.068
	Yes (1-3 days/week)	26	2 (0-23)		2 (0-20)		20 (7-28)	
	Yes (4-5 days/week)	33	5 (0-15)		4 (-013)		22 (7-32)	
	Yes (6-7 days/week)	50	4.5 (0-14)		2.5 (0-18)		19 (10-31)	

We explored the factors reported to be most troubling for the participants. Following multiple responses analysis, taking over school lessons was found to be the most troubling factor affecting the participants' level of distress during COVID-19, followed by working from home and worrying about the health of their family members. The detailed results are depicted in Table 3.

**Table 3:** Rank of the psychosocial factors that are most reported to cause distress by the participants

Factors that are reported to be troubling the participants		N (%) of total respondents who answered yes
F9	Taking over school lessons at home	53 (63.9)
F8	Working from home	47 (56.6)
F2	Worries about the health of my family members	39 (47.0)
F3	Financial worries	27 (32.5)
F4	Difficulties to adapt to the new norms	25 (30.1)
F5	Uncertainties regarding my job	25 (30.1)
F1	Worries about my health	23 (27.7)
F6	Increased conflicts with people close to me	16 (19.3)
F7	Being in quarantine	14 (16.9)

We further analyze the association between the psychosocial factors and the level of depression, anxiety, and FOC (Appendix 3). The results of the analysis are presented in Table 4. From the study, we found several significant psychosocial factors that were associated with depression, which are: worries about one's health (p = 0.002), worries about family's health (p = 0.009), financial worries (p≤0.001), difficulties adapting to new norms (p = 0.028), being in quarantine (p = 0.002), working from home (p = 0.002), and taking over school lessons (p = 0.032). Meanwhile, increased conflict with people close to me is not associated with depression (p = 0.949).

**Table 4:** Spearman correlation between psychosocial factors with the level of depression, anxiety, and FOC *p<0.05, statistically significant FOC: Fear of COVID-19

Psychosocial Factors (F)	Participants’ response	Depression level			Anxiety level			FOC		
		Normal to mild	Moderate to severe	p-value	Normal to mild	Moderate to severe	p -value	Low fear	High fear	p -value
F1 My own health	Yes, N (%)	13 (56.5)	10 (43.4)	0.002*	19 (82.6)	4 (17.4)	0.125	10 (43.5)	13 (56.5)	0.048*
	No, N (%)	64 (86.5)	10 (13.5)		69 (93.2)	5 (6.8)		47 (63.5)	27 (36.5)	
F2 My family’s health	Yes, N (%)	27(69.2)	12 (30.8)	0.009*	34 (87.2)	5 (12.8)	0.072	19 (48.7)	20 (51.3)	0.151
	No, N (%)	54 (90.0)	6 (10.0)		58 (96.7)	2 (3.3)		38 (63.3)	22 (36.7)	
F3 Financial worries	Yes, N (%)	14 (52.0)	13 (48.0)	<0.001*	20 (74.1)	7 (25.9)	<0.001*	15 (55.6)	12 (44.4)	0.181
	No, N (%)	66 (90.4)	7 (9.6)		72 (98.6)	1 (1.4)		44 (60.3)	29 (39.7)	
F4 Difficulties to adapt to the new norms	Yes, N (%)	16 (64.0)	9 (36.0)	0.028*	21 (84.0)	4 (16.0)	0.081	12 (48.0)	13 (52.0)	0.166
	No, N (%)	65 (84.4)	12 (15.6)		73 (94.8)	4 (5.2)		49 (63.6)	28 (36.4)	
F5 Uncertainties of the future	Yes, N (%)	15 (60.0)	10 (40.0)	0.004*	20 (80.0)	5 (20.0)	0.026*	12 (48.0)	13 (52.0)	0.157
	No, N (%)	65 (86.7)	10 (13.3)		71 (94.7)	4 (5.3)		48 (64.0)	27 (36.0)	
F6 Conflicts with people close to me	Yes, N (%)	13 (81.2)	3 (18.8)	0.949	15 (93.8)	1 (6.3)	0.889	12 (75.0)	4 (25.0)	0.264
	No, N (%)	68 (81.9)	15 (18.1)		77(92.8)	6 (7.2)		50 (60.2)	33 (39.8)	
F7 Being in quarantine	Yes, N (%)	31 (66.0)	16 (34.0)	0.002*	12 (85.7)	2 (14.3)	0.263	7 (50.0)	7 (50.0)	0.283
	No, N (%)	51 (91.1)	5 (8.9)		65 (94.2)	4 (5.8)		45 (65.2)	24 (34.8)	
F8 Working from home	Yes, N (%)	31 (66.0)	16 (34.0)	0.002*	40 (85.1)	7 (14.9)	0.013*	27 (57.4)	20 (42.6)	0.275
	No, N (%)	51 (91.1)	5 (8.9)		55 (98.2)	1 (1.8)		38 (67.9)	18 (32.1)	
F9 Taking over school lessons	Yes, N (%)	41 (77.4)	12 (22.6)	0.023*	47 (88.7)	6 (11.3)	0.032*	30 (56.6)	23 (43.4)	0.530
	No, N (%)	36 (94.7)	2 (5.3)		38 (100)	0 (0)		24 (63.2)	14 (36.8)	

The study reveals a statistically significant association of psychosocial factors with moderate to severe anxiety, which includes financial worries (p<0.001), uncertainties about the future (p = 0.026), working from home (p = 0.013), and taking over school lessons (p = 0.032). Meanwhile, worry about one's health was associated with a high FOC (p = 0.048). In contrast, no psychosocial factors were significantly related to FOC. 

The Pearson correlation matrix shows that the strongest correlation of 0.777 exists between factor 5 (uncertainties regarding my job) and factor 9 (taking over school lessons), as shown in Table 5. This is followed by concerns about personal health (factor 1) and family health (factor 2). Factor 9 was also correlated with several factors (factor 1, factors 2 to 7), which confirmed that it was a considerable concern among the respondents.

**Table 5:** Pearson correlation matrix of the studied psychosocial factors causing distress Correlation is significant at the 0.01 level (2-tailed) * signifies significance

	Psychosocial Factors	F1	F2	F3	F4	F5	F6	F7	F8	F9
F1	Worries about my health	1	-	-	-	-	-	-	-	-
F2	Worries about my family’s health	0.737*	1	-	-	-	-	-	-	-
F3	Financial worries	0.577	0.553	1	-	-	-	-	-	-
F4	Difficulties in adapting to the new norms	0.554	0.455	0.561	1	-	-	-	-	-
F5	Uncertainties regarding my job	0.639	0.478	0.652	0.675	1	-	-	-	-
F6	increased conflicts with people close to me	0.336	0.320	0.382	0.493	0.492	1	-	-	
F7	Being in quarantine	0.416	0.390	0.490	0.610	0.529	0.455	1	-	-
F8	Working from home	0.296	0.313	0.433	0.322	0.350	0.268	0.438	1	-
F9	Taking over school lessons	0.5838*	0.402*	0.623*	0.639*	0.777*	0.536*	0.564*	0.350	1

## Discussion

This study showed several interesting findings. In contrast to previously published studies that found a higher risk of anxiety, depression, and FOC among females during the initial phase of the outbreak, this study found that the prevalence was equal among both genders [1-3]. Similar findings were reported by Dai et al. [37]. This could be attributed to the findings of a recent study that although 70% of COVID-19 admissions in Malaysia were male, the risk of having severe COVID-19 infection was equal for both genders [38].

This study also found no significant association between levels of anxiety and depression, and other demographic characteristics, such as age group and marital status. However, existing literature has highlighted that younger adults experienced higher levels of depression and anxiety during the pandemic [39]. Furthermore, a significant relationship was observed between relationship status and psychological distress, indicating that single individuals reported the highest levels of depressive and anxiety symptoms during the pandemic [40].

The current study found a statistically higher FOC among non-academic employees. Unlike academicians, whose main challenges were transforming their teaching and academic-related activities into virtual education, some of the administrative and non-academic duties may needed to be accomplished physically, which indirectly increased the exposure to COVID-19, resulting in heightened FOC among non-academic employees [41]. A significantly higher FOC was found among the permanent NDUM employees than contract workers. We postulated that the permanent employees experienced a higher level of fear as they are bound to the workplace regulations to maintain their work performance amidst the difficulties and the risk of COVID-19 exposure faced during the pandemic.

This study explored the psychosocial factors that were believed to be associated with psychological distress during the phase of MCO. Taking over school lessons was identified as the most problematic for our participants, and it was significantly associated with having depressive and anxiety symptoms. This finding resonated with the previously published paper identifying several factors contributing to parental stress during the pandemic [42]. The MCOs imposed during COVID-19 led to drastic shifts in parental roles. The closures of childcare facilities and schools have forced working parents to multitask at home, assuming the role of their children’s educators while concurrently working and performing domestic responsibilities. Parents were expected to provide appropriate electronic devices suitable for virtual classes. Hence, there had been unexpected extra financial expenses, monitoring the school lessons and updating the arrangement for virtual classes and online school activities for their children [43]. In a recent study among Malaysian parents, four significant adverse impacts of homeschooling during COVID-19 were identified, which include low academic performance, poor concentration, lack of social interaction, and stress-related behavior [44]. Undeniably, these challenges can lead to parental distress [45].

This factor further explained another finding that working in their respective home office was identified as the second factor to be the concern to our participants, which affected their mental health. Although working from home has advantages such as eliminating commuting time, reducing travel costs, and providing more flexibility, it has been shown that working from home can harm an individual's psychological state and productivity [46]. A study by Xiao et al. shows that remote work can negatively affect psychological health due to increased distractions, changes in communication with co-workers, and alterations in working hours [47]. Workers who were working remotely from home during the pandemic were also found to have higher levels of loneliness and negative emotions [48].

Our study confirmed that the uncertainties about the future and financial worries were associated with a negative psychological state during the COVID-19 pandemic. In a survey among university employees in Brazil, Serralta et al. found that the prospect of losing their job and income was reported by 58% of the respondents, followed by loneliness and changes in their personal lives and routines [49]. In Malaysia, the unemployment rate reached 4.5% in 2020, the highest in three decades [50]. This figure may have triggered uncertainties about future and possible job losses, even among university employees, as observed in Australia [51].

Our study found that worries about one’s health and the health of family members were associated with depression. This was likely due to selected public general hospitals being transformed into dedicated COVID-19 treatment centers and healthcare workers being reallocated throughout the country, leading to disruptions and delays in healthcare services for chronic diseases and surgical procedures during the pandemic [52,53]. Furthermore, a recent review of the epidemiology of COVID-19 in Malaysia found that 87.3% of COVID-19 deaths were among individuals aged 50 years and older [54]. It is not surprising that the healthcare constraints and high mortality rate during the pandemic contributed to fears regarding one’s health and the health of family members, as well as psychological distress. A study in the United States showed that delays in surgical and dental care, particularly among the middle-aged and elderly populations, contributed to symptoms of depression and anxiety [55].

**Figure 4:**
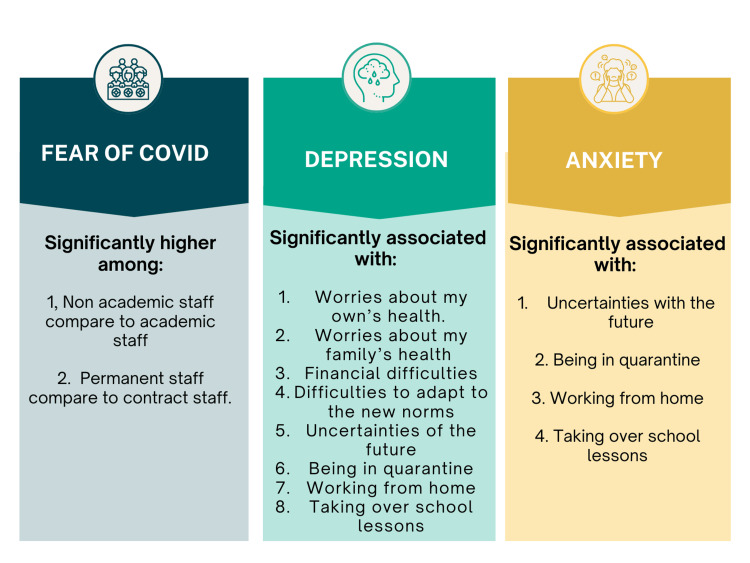
Principal findings of this research paper This figure has been drawn utilizing the Canva premium version (https://www.canva.com/) [25]. Image Credit: Fatimah Zahra Mohamad Rom.

The strength of this study is that, to the best of our knowledge, it is the first study in Malaysia to explore the psychosocial factors deemed essential and causing distress during the isolation period. While most of the studies during COVID-19 explored the impact of mental health on essential workers, this study helps to fill the gap by assessing the psychological impact of the pandemic on workers of other sectors, particularly university staff. The principal findings of this paper were FOC followed by depression and anxiety, illustrated in Figure 4.

Study limitations 

This research used a cross-sectional design within a single higher education institution, limiting the findings' generalizability to the broader Malaysian workforce. With a response rate of 46.2%, the views expressed may not fully represent those of all NDUM employees, which could lead to response bias. While this low engagement may limit the generalizability of the findings, it is crucial to acknowledge the unique circumstances affecting participants. The restrictions imposed during the MCO likely impacted individuals' ability and willingness to participate, compounded by the stress and uncertainty associated with the pandemic, especially within the academic sector. Despite these challenges, the data collected offer valuable insights into the psychological effects of lockdown measures on university employees and the shift to distance learning. Future research should explore strategies to enhance participant engagement, such as targeted outreach or incentives, to improve response rates and obtain a more representative sample. Finally, the study's cross-sectional nature prevented us from determining causal relationships between psychological symptoms and the associated psychosocial factors.

Future studies****

Future studies should include multiple higher education institutions in Malaysia to enhance the representativeness of the results for university employees. The current study’s results highlight an urgent need for universities to prioritize mental health initiatives that address these specific psychosocial stressors. The findings from this study implicate the importance of psychological distress experienced by university employees during the pandemic. This distress is associated with various psychosocial factors which can severely impact mental health. Understanding these factors allows institutional leaders and policymakers to create tailored support systems to mitigate distress and foster a healthier work environment.

Future studies should also explore targeted interventions and assess their effectiveness in reducing distress among affected employees. For instance, research could investigate the impact of mental health training programs, peer support networks, and flexible work arrangements on employee well-being. We recommended future research initiatives to prevent such mental health issues based on our study findings. Several practical strategies should be implemented to prepare for and prevent mental health challenges. The necessary programs must be put into practice, e.g., institutional mental health policies, proactive mental health initiatives, and regular mental health assessments among university employees. Furthermore, authorities must be committed to establishing feedback mechanisms where employees are able to express their mental health concerns freely, and university authorities should take necessary action.

## Conclusions

Our findings indicate significant psychological distress symptoms among employees. Although the low response rate limits the generalizability of these results, several critical psychosocial factors contributing to distress among NDUM university staff were identified. These outcomes highlight the crucial need for additional research into these psychosocial factors to effectively address mental health challenges during pandemics. In light of these findings, we strongly advocate for collaboration among institutional leaders, policymakers, and university communities to implement proactive measures to enhance and support the mental health and well-being of university employees.

## Appendices

Appendix 1 

**Table 6:** PHQ-9 [28,29] PHQ-9: Patient Health Questionnaire-9

Over the last 2 weeks, how often have you been bothered by any of the following problems?	NOT AT ALL	SEVERAL DAYS	MORE THAN HALF THE DAYS	NEARLY EVERY DAY
Little interest or pleasure in doing things	0	1	2	3
Feeling down, depressed, or hopeless	0	1	2	3
Trouble falling or staying asleep, or sleeping too much	0	1	2	3
Feeling tired or having little energy	0	1	2	3
Poor appetite or overeating	0	1	2	3
Feeling bad about yourself – or that you are a failure or have let yourself or your family down	0	1	2	3
Trouble concentrating on things, such as reading the newspaper or watching television	0	1	2	3
Moving or speaking so slowly that other people could have noticed. Or the opposite – being so fidgety or restless that you have been moving around a lot more than usual	0	1	2	3
Thoughts that you would be better off dead, or of hurting yourself in some way	0	1	2	3

Appendix 2 

**Table 7:** GAD-7 scale [30,31] GAD-7: Generalized Anxiety Disorder-7

Over the last 2 weeks, how often have you been bothered by the following problems?	Not at all sure	Several days	Over half the days	Nearly every day
1. Feeling nervous, anxious, or on edge	0	1	2	3
2. Not being able to stop or control worrying	0	1	2	3
3. Worrying too much about different things	0	1	2	3
4. Trouble relaxing	0	1	2	3
5. Being so restless that it's hard to sit still	0	1	2	3
6. Becoming easily annoyed or irritable	0	1	2	3
7. Feeling afraid as if something awful might happen Add the score for each column Total Score (add your column scores) =	0	1	2	3
	+	+	+	

Appendix 3 

**Table 8:** FOC This questionnaire was developed utilizing the study by Ahorsu et al. [32]. Scoring: The participants indicate their level of agreement with the statements using a five-item Likert-type scale. Answers included “strongly disagree,” “disagree,” “neutral,” “agree,” and “strongly agree.” The minimum score possible for each question is 1, and the maximum is 5. A total score could be calculated by adding up each item score (ranging from 7 to 35). FOC:  Fear of COVID-19

No of Questions	Questions	﻿Strongly Disagree	Disagree	Neutral	Agree	Strongly Agree
	﻿I am most afraid of Corona.					
	﻿It makes me uncomfortable to think about Corona.					
	﻿My hands become clammy when I think about Corona.					
	﻿I am afraid of losing my life because of Corona.					
	﻿When I watch news and stories about Corona on social media, I become nervous or Anxious.					
	﻿I cannot sleep because I’m worrying about getting Corona.					
	My heart races or palpitates when I think about getting Corona					
